# Confirmatory factor analysis of the Evidence-Based Practice Attitude Scale (EBPAS) in a large and representative Swedish sample: is the use of the total scale and subscale scores justified?

**DOI:** 10.1186/s12874-020-01126-4

**Published:** 2020-10-14

**Authors:** Anna Helena Elisabeth Santesson, Martin Bäckström, Robert Holmberg, Sean Perrin, Håkan Jarbin

**Affiliations:** 1 Department of Clinical Sciences, Faculty of Medicine, Lund University, BMC F12, S-, 221 84 Lund, Sweden; 2 Department of Psychology, Faculty of Social Sciences, Lund University, Lund, Sweden

**Keywords:** Attitudes, EBP, Evidence-based practice attitude scale (EBPAS), Implementation, Validation, Psychometric evaluation, Psychometric properties, Confirmatory factor analysis (CFA), Bifactor model

## Abstract

**Background:**

There is a call for valid and reliable instruments to evaluate implementation of evidence-based practices (EBP). The 15-item Evidence-Based Practice Attitude Scale (EBPAS) measures attitude toward EBP, incorporating four lower-order factor subscales (Appeal, Requirements, Openness, and Divergence) and a Total scale (General Attitudes). It is one of a few measures of EBP attitudes evaluated for its psychometric properties. The reliability of the Total scale has been repeatedly supported, but also the multidimensionality of the inventory. However, whether all of the items contribute to the EBPAS Total beyond their subscales has yet to be demonstrated. In addition, the Divergence subscale has been questioned because of its low correlation with the other subscales and low inter-item correlations. The EBPAS is widely used to tailor and evaluate implementation efforts, but a Swedish version has not yet been validated. This study aimed to contribute to the development and cross-validation of the EBPAS by examining the factor structure of t a Swedish-language version in a large sample of mental health professionals.

**Methods:**

The EBPAS was translated into Swedish and completed by 570 mental health professionals working in child and adolescent psychiatry settings spread across Sweden. The factor structure was examined using first-order, second-order and bifactor confirmatory factor analytic (CFA) models.

**Results:**

Results suggested adequate fit for all CFA models. The EBPAS Total was strongly supported in the Swedish version. Support for the hierarchical second-order model was also strong, while the bifactor model gave mixed support for the subscales. The Openness and Requirements subscales came out best, while there were problems with both the Appeal (e.g. not different from the General Attitudes factor) and the Divergence subscales (e.g. low reliability).

**Conclusions:**

Overall, the psychometric properties were on par with the English version and the total score appears to be a valid measure of general attitudes towards EBP. This is the first study supporting this General Attitudes factor based on a bifactor model. Although comparatively better supported in this Swedish sample, we conclude that the use of the EBPAS subscale scores may result in misleading conclusions. Practical implications and future directions are discussed.

## Background

Health practitioner’s attitudes and values play an important role in implementing evidence-based practice (EBP) in community care settings [1]. Positive attitudes along with subjective norms and a person’s self-efficacy may influence the practitioner’s decision whether or not to implement a new practice [2, 3]. According to Rogers, the innovation-decision process starts with knowledge and persuasion when the provider [practitioner] forms a favorable or unfavorable attitude toward the innovation [4]. Both general and specific constructs play an important role in understanding and predicting behavior. General predictor variables are suggested as the best to predict general outcomes and specific predictor variables the best to predict specific outcomes [5, 6]. Assessing both specific and general attitudes towards EBP may help in the tailoring and evaluation of an implementation program [7].

Psychometrically sound and theory-based measurements, including those measuring attitude towards EBP, are essential for the field of implementation science [8, 9]. A good scale consists of a heterogeneous set of items that capture the entire breadth of a given construct while providing acceptable reliability. A scale’s dimensionality can have important consequences for scale scoring and interpretation [6]. Although only valid and reliable measures can confidently and consistently measure what they are intended to measure, psychometric information is absent in about half of all articles using various scales as part of innovation implementation programs in healthcare settings [8, 10]. Other problems are the lack of short and pragmatic instruments and instruments with a broad application used across studies [11].

The Evidence-Based Attitude Practice Scale (EBPAS) is an instrument of high overall psychometric quality [12]. The EBPAS was developed by Aarons on the basis of a comprehensive literature review and consultation with mental health service providers and researchers. Fifteen items generate a total score and subscale scores covering four important domains of attitudes toward EBP: the intuitive *Appeal* of EBP to the provider; response to organizational *Requirements*; *Openness* to new/manualized interventions; and perceived *Divergence* [1, 13, 14]. Subscale scores are used to obtain information about the specific domains of attitude toward EBP and the total score (where the Divergence items are reversed) is used to estimate a common dimension of global attitude to EBP. Previous studies have suggested good internal consistency for the total scale and the subscale scores except for the Divergence subscale [1, 13, 14].

The convergent validity has been examined by correlation with theoretically related constructs, supporting the total scale and to some extent the subscales [15–18]. Consistent with expectation, EBPAS total and subscales scores positively correlate with provider education levels, leadership quality and attitudes towards change, and the implementation climate [14, 19–22]. In respect of concurrent validity, attitudes toward EBP, measured by the EBPAS, are expected to have a relationship to service delivery [22]. Previous studies find that scores on the Openness subscale are positively correlated with self-reported use of manuals and cognitive-behavioral therapy [23, 24], while higher scores on the Divergence subscale are associated with the use of non-evidence-based therapy strategies [24]. However, no such associations were found between usage and scores on the Appeal and Requirements subscales; and correlations between practice indicators and the EBPAS total score were not tested [23, 24]. In further support of the concurrent validity of the EBPAS, one study found that psychotherapists who scored higher on the Openness scale prior to training in EBP reported more fidelity consistent modifications at follow-up and those who scored higher on the Appeal subscale reported more fidelity inconsistent modifications [25]. Subsequent studies have also found that higher Requirement scores, but not scores on the other subscales, are associated with non-adherence, non-skillful usage, and non-usage of EBP techniques after training [26, 27]. Staff turnover is a major problem in mental health settings and may jeopardize implementation and sustainment of EBP [28]. Higher scores on the Openness subscale predicted greater workplace retention [28]. Practitioners who scored higher on the Divergence subscale had a higher likelihood of EBP discontinuation, another threat to EBP sustainment [29]. The latter study used only the Divergence and Openness subscales and no association between Openness subscale scores and EBP discontinuation was found.

The construct validity of the EBPAS has been investigated in several studies using Confirmatory factor analysis (CFA) [1, 13, 14] (Additional file 1). The fit of first-order models support the placement of the EBPAS items in four separate subscales, with moderate to strong factor loadings for all subscales except Divergence [1, 13, 14]. The factor loadings have generally been moderate to strong with the weakest loadings for the Divergence scale. Correlations between the factors have generally been moderate, suggesting that they in part measure the same overall construct, but also here, the Divergence scale has shown weak correlations with some of the other scales [1, 13]. To remedy the lack of support for the Divergence scale, a more complicated five-factor model has been tested wherein the Divergence subscale is split in to two factors [30], but subsequent research did not support this model [31]. The hypothesis of a general attitude factor using all 15 items (i.e. a total score) from the EBPAS has been supported by acceptable model fit for a second-order model [14]. The first-factor loadings were strong and loadings on the general factor moderate to strong. Again the Divergence scale has been least supported. Items from this subscale had inconsistent loadings on the Divergence factor itself and the Divergence factor had a weak loading on the general factor [14]. Results from one bifactor model study provided preliminary support for the hypothesis that the variance in the individual EBPAS items can be attributed to a general factor and uniquely to the four domain-based factors (Appeal, Requirements, Openness, Divergence) [32] (Additional File 1). In that study, the bi-factor model had a slightly better fit compared to the second-order model with significant first-factor loadings in the moderate to strong range (with an exception for the Appeal subscale). Factor loadings or the general attitudes (total) scale were moderate except for items from the Divergence subscale, which were weak or non-significant.

In sum, the available research provides preliminary support for the proposed factor structure (four subscales and a total scale) for the EBPAS, but several issues remain. The model has been revised such that two items in the Appeal subscale were allowed to correlate, reflecting the possibility that these two items have more in common than can be accounted for by the subscale itself (e.g. they may suggest an additional specific factor) [13, 14]. Furthermore, across studies, there are clear difficulties with the factor loadings for the Divergence subscale [1, 13, 14, 30, 32].

The EBPAS has been translated into different languages and cross-validated in various settings, however a Swedish version has not yet been validated [30–36]. The present study aimed to help fill this gap in the literature. Studies assessing the factorial validity of translated scales are important because they provide further evidence of the cross-cultural validity of the constructs assessed by that scale. New problems can be the results of a translation and it is important to show that the translation does not result in weaker support for the scale’s validity.

To summarize, the EBPAS, or parts of it, is widely used in implementation research. Previous studies give some support for its construct validity especially the general factor. There is less support for the EBPAS as a multidimensional scale with four subscales contributing uniquely to the general attitude construct. In other words, is it meaningful to include all of the sub-factors in the EBPAS general factor? Is it wise to add items/subscales together into a total scale or use the subscales independently as indicators of specific attitude towards EBP or predictors for other implementation outcomes?

The present study aimed to address these gaps in the literature. The construct validity of a Swedish version of the EBPAS was examined in a large and representative sample of practitioners working in child mental health settings across Sweden thus following previous studies by the scale developer [1, 13, 14]. In addition we conducted a confirmatory bifactor analysis to evaluate the plausibility of scoring and using the subscales [32]. Specifically, this study aimed to investigate: 1) the reliability of a Swedish translation of the EBPAS; 2) its factorial structure relative to previous (first-, second-order and bifactor models) tested in English and non-English version of this scale and 3) whether sub-factors are uniquely supported in the Swedish version of the inventory, in other words, whether the subscale domains are supported when the general attitude domain has been accounted for?

## Methods

### Design and setting

Data from the current cross-sectional study were obtained as part of a large prospective, multi-center implementation study of evidence-based interventions for depressed youth. All publicly (state) funded child and adolescent mental health services (CAMHS) in Sweden were invited to participate in the Swedish Association of Child and Adolescent Psychiatry “Deplyftet” implementation program for youth depression. Individual Swedish CAMHS serves about 64,000 children annually (range = 29,000- 450,000) children. The current study uses a subsample of data drawn from providers working at 11 of 31 eligible CAMHS who collectively serve about 712,000 youth (36% of all Swedish children). The individual CAMHS from which the current data are drawn represent all types of publicly owned and funded CAMHS, serving similar-sized catchment areas as the remaining CAMHS (i.e., average = 65,000 youth, range = 41,000- 125,000).

### Procedure

The validation of the Swedish version of the EBPAS was conducted in two stages. First, the EBPAS was translated from English into Swedish by an expert group of mental health professionals following recommendations for the translation of measures (see below) [36]. Second, the EBPAS was administered to 925 professionals working in Swedish CAMHS via a web-based survey. Data were collected as a baseline assessment from October 2014 to February 2017 with 2–5 reminders sent (if necessary) and the resulting data used to examine the psychometric properties of the EBPAS.

### Measure

The web-based survey included questions about the respondent’s age, gender, and professional background, followed by the EBPAS.

#### The EBPAS

The EBPAS consists of 15 items rated on a Likert scale (0 = *not at all* to 4 = *to a very great extent)* and is comprised of four subscales: 1) *Appeal* (four items) measures the intuitive appeal of the EBPs; 2) *Requirements* (three items) measures the extent to which the provider would adopt a new practice if it were required; 3) *Openness* (four items) measures the extent to which the provider is generally willing to try new interventions; and 4) *Divergence* (four items) measures the extent to which the provider perceives research based treatments as not clinically useful and/or less important than their own clinical experience [1]. Requirement differs from Openness in that the former assesses how employees respond to organizational rules and regulations, while the latter measures the extent to which the provider is generally willing to try new interventions. Previous studies report Cronbach alphas ranging from .76 to .91, except for the Divergence scale (.59 -to .66) [1, 13, 14].

### Cross cultural adaptation and translation

Permission to translate the EBPAS was obtained from the scale’s developer [1]. Item 13 (Requirement subscale) was adapted for the Swedish context while preserving the integrity of the original item. The word “State” in item 13 was replaced with “National Board of Health and Welfare.” All other items were simple translations of the original. A step-wise forward-backward translation approach was utilized [37]. The EBPAS was translated (separately) into Swedish by the first and last authors (AS, HJ), who are native Swedish speakers and fluent in English. The two translations were compared, discrepancies identified, and any discrepancies or deviations from the original item were resolved and a final Swedish version produced. This Swedish version was then back-translated into English by a professional translator. The first two authors compared the back-translated version to the English language original and the final Swedish version. No further changes were necessary. The final back-translated version was reviewed and approved by the scale developer.

### Psychometric testing

A series of CFAs were conducted to test the factor structure as the EBPAS has been thoroughly examined in previous studies using both exploratory and confirmatory methods [13, 14]. We tested three models, all specified a priori, using the entire sample for each model: 1) a four factor model based on the suggested subscales of the inventory; 2) a higher- order model, with one General Attitudes factor on the level above the four subscales; and 3) a bifactor model measuring a General Attitudes factor defined to be unrelated to the sub-factors (this model was used to test for unique variance of the four scales to the general attitudes to EBP construct). The problem with first-order models is that they do not explicitly support the general factor, e.g. a sum score of all included scales [6]. In second-order models, each item loads on their specific factors, and all sub-factors load on a higher-order construct that accounts for the commonality between sub-factors. Bifactor models are an alternative to the second-order models with the advantage that it is possible to test for unique contribution of the sub-factors. In bifactor models individual items load on both a general factor and a specific factor. In other words, bifactor models test whether there is support for a specific factor after accounting for the general factor [38, 39].

### Participants

A total of 570 (62%) of the 925 outpatient practitioners working in the 11 CAMHS responded to the online survey. Of these, five were excluded; three because of missing data for all items, one because all responses were the same, and one for being a multivariate outlier (Mahalanobis distance with *p* < 0.001), leaving 565 participants for analysis. Two univariate outliers (extremely high *z* scores > 3.0) were replaced with the same value as their closest neighbors [40]. The typical participant was female (84%), 35–45 years old (28%) and a psychologist (38%).

### Statistical analyses

Descriptive statistics, item-total correlation and internal consistency reliability (Cronbach α) were analyzed using SPSS (Version 24) [41]. CFA models were estimated using MPLUS 8 [42]. The weighted least squares -robust mean and variance adjusted (WLSMV) estimator was used since the items were ordinal (Likert scale). Cases with missing data were included in the CFA because the WSLW estimator permits their inclusion. Several different model fit indices were used:; chi-squared index (χ^2^), the comparative fit index (CFI), the root mean square error of approximation (RMSEA), and the standard root mean square residual (SRMR). As an alternative estimation we tested the models with robust maximum likelihood (MLR), using this estimation the RMSEA revealed much better fit. RMSEA has been shown to be problematic together with WLSMV estimation [43]. For categorical models Yu has recommended .95 for CFI, .05 for RMSEA and for SRMR a good model should have lower than .08 [44]. …. In addition, we evaluated the explained common variance (ECV) for the bifactor model to evaluate the importance of the General Attitudes factor in comparison to the four subscale -factors [6]. We also estimated the variance for the EPBAS scales that could be attributed to the services using the intra class coefficient (ICC). All the ICC was found to be low, the highest was .021 for the Requirement subscale, and the ICC for the total scale was .020. A sample of 300 is sufficient for conducting CFAs [40].

## Results

### Descriptive item analysis

Table 1 presents the descriptive statistics for the individual items, subscales and total scale. Mean values were high for most of the positively phrased EBPAS items and generally lower for negatively worded items. Skewness was generally negative (J-shaped) for positively phrased items, but above 1.0 only for one item. Three items had a kurtosis value > 1. Item 15 (enough training) was extremely kurtotic (2.6) with a positive skew > 1.0. This item also had a very obvious ceiling effect with 54% of the ratings in the highest response category.

**Table 1:** EBPAS subscale and item means, standard deviations, Cronbach’s alpha and item-total correlation

EBPAS subscales and Total				Total scale	Sub-scale
	*M*	*SD*	*α*	*r*	*r*
Requirements	2.70	0.82	.88		
12.Agency required	2.61	0.92		.49	.88
11.Supervisor required	2.51	0.97		.44	.81
13.State required	2.98	0.84		.48	.63
Appeal	3.24	0.52	.74		
10.Make sense	3.24	0.66		.43	.61
9.Intuitively appealing	3.15	0.75		.44	.58
14.Colleagues happy with therapy	3.11	0.72		.43	.46
15.Enough training	3.47	0.66		.53	.48
Openness	2.88	0.58	.76		
2.Will follow a treatment manual	2.98	0.78		.58	.68
4.Therapy developed by researchers	3.08	0.72		.55	.59
1.Like new therapy types	2.90	0.72		.31	.52
8.Therapy different than usual	2.58	0.80		.39	.47
Divergence	1.20	0.59	.60		
5.Research-based treatments not useful	0.92	0.92		.30	.42
7.Would not use manualized therapy	0.67	0.88		.53	.36
6.Clinical experience more important	1.90	0.84		.34	.42
3.Know better than researchers	0.28	0.93		.35	.27
EBPAS total	2.92	0.42	.81		

*N* = 565. Total, subscales and item means scores range from 0 to 4. *α* Cronbach alpha, *r* Corrected item total

### Reliability

Internal consistency was .81 for the total scale and ranged from α = .60 to .88 for the subscales (Table 1). Item-total correlations ranged from .30 to .58 for the total scale and from .27 to .88 for the subscales. No improvements in Cronbach’s alphas occurred with removal of individual items.

### Construct validity

**Fig. 1:**
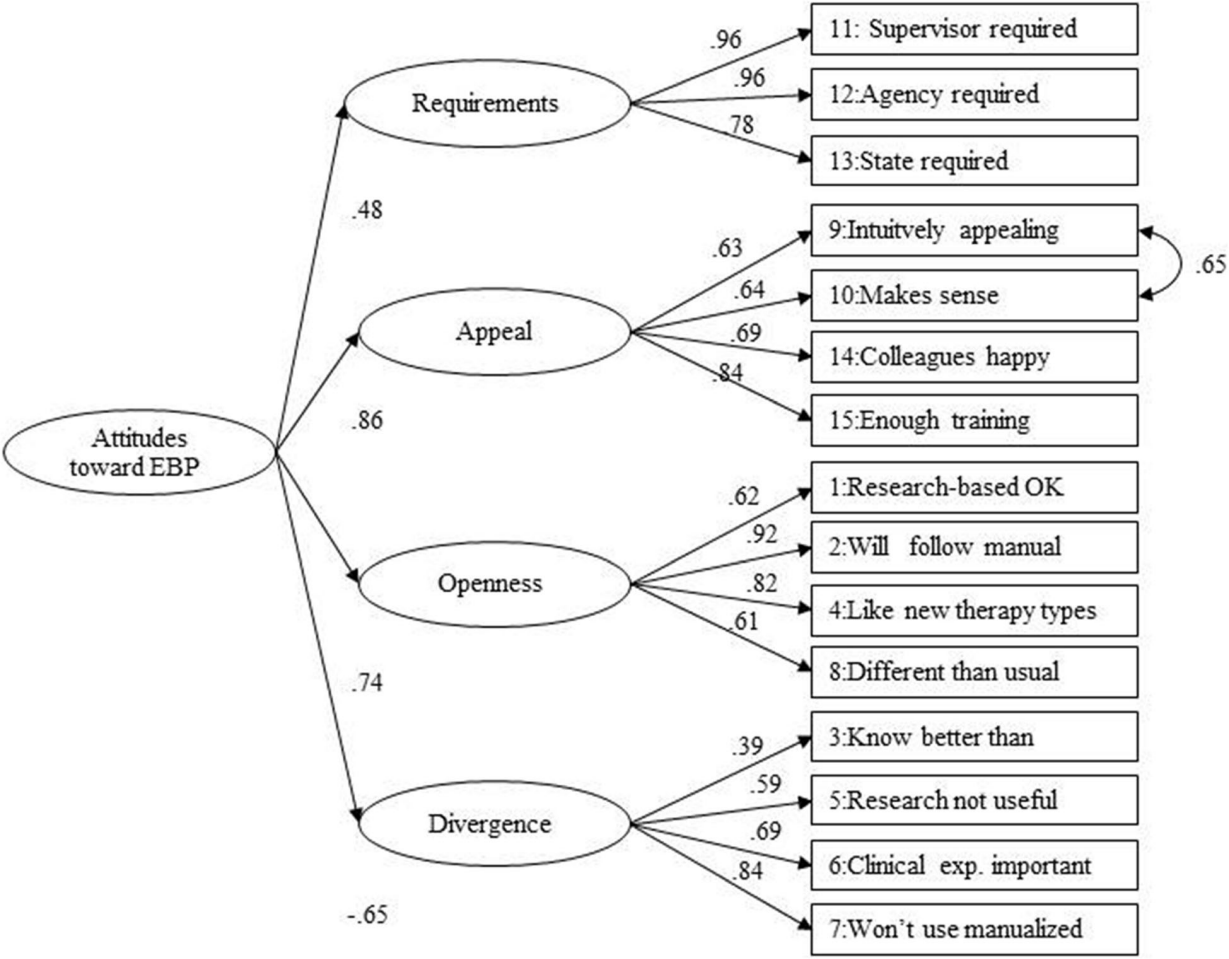
Second-Order Confirmatory factor analysis model. Standardized factor loadings for model 2b, *n* = 565, χ^2^ (86) =558.5, CFI = .973, RMSEA = .098, SRMR = 0.006. Estimation of residuals between Appeal subscale items is indicated by a double-headed arrow. All factor loadings are significant at the *p* < .001 level

**Fig. 2:**
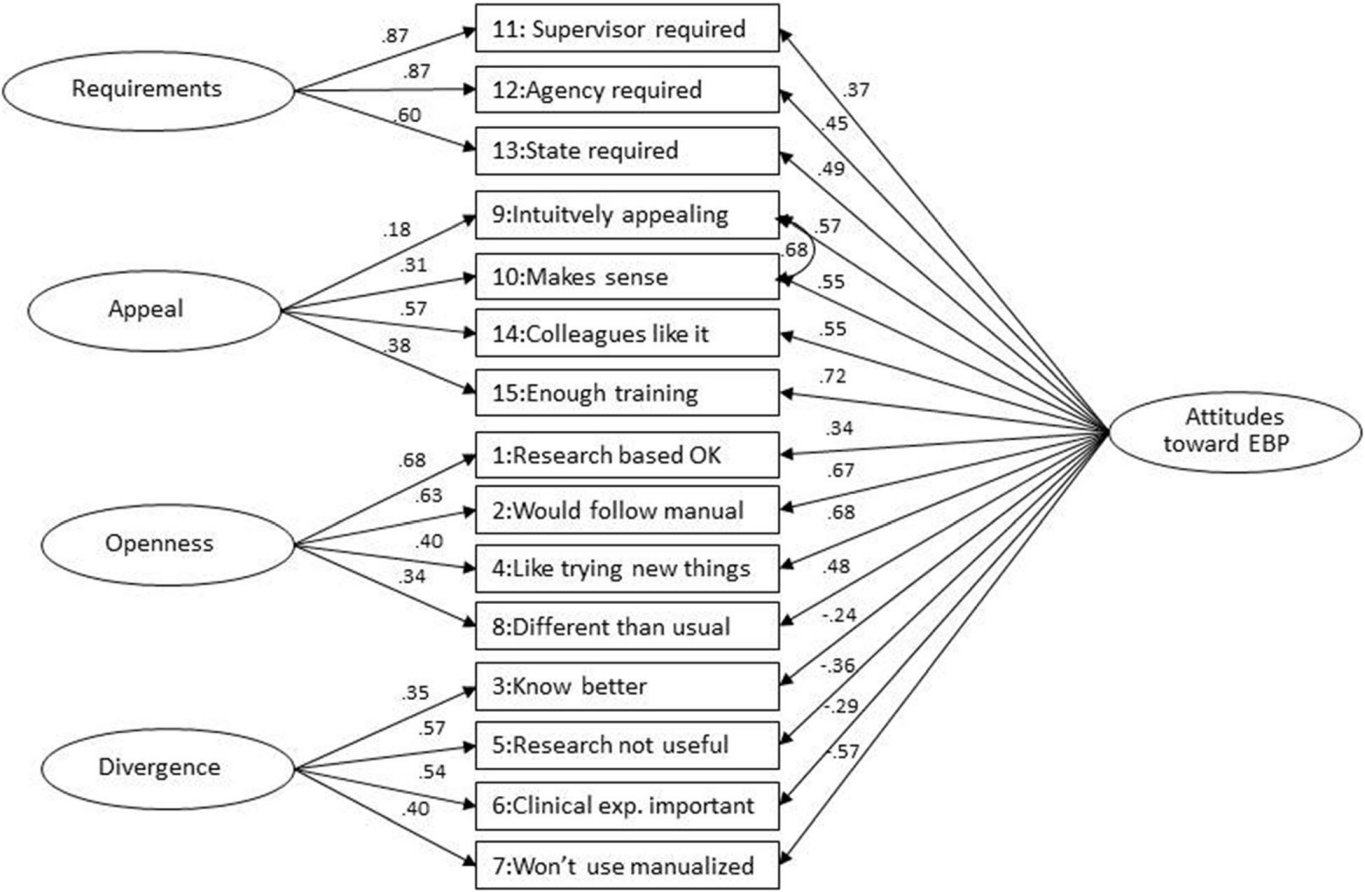
Bifactor Model Standardized factor loadings for model 3, n = 565, χ^2^ (75) =450.5, CFI = .978, RMSEA = .094, SRMR = 0.058. Estimation of residuals between Appeal subscale items is indicated by a double-headed arrow. All factor loadings are significant at the *p* < .001 level

The model fit indices are presented in Table 2, the factor inter-correlations in Table 3 and the factor loadings in Table 4. For the second-order and bifactor models, the factor loadings are also presented in Figs. 1 and 2, respectively.

**Table 2:** EBPAS domain correlations

	Requirement	Appeal	Openness	Divergence
Requirement				
Appeal	.545			
Openness	.251	.612		
Divergence	−.217	−.398	−.612	

Numbers are Pearson’s correlation coefficient. All correlations are significant

**Table 3:** Model fit information for five alternative models of the EBPAS (*N* = 565)

Model	χ^2^	Df	*p*	CFI	RMSEA	SRMR
First-order (1a)	555.1	85	<.001	.973	.099	0.061
First-order (1b)	399.0	84	<.001	.982	.081	0.053
Second-order (2a)	687.5	87	<.001	.965	.110	0.072
Second-order (2b)	558.5	86	<.001	.973	.098	0.066
Bifactor (3)	450.5	75	<.001	.978	.094	0.058

**χ**^2^ = Chi-square index, *Df* Degrees of freedom, *CFI* Comparative fit index, *RMSEA* Root mean square error of approximation and *SRMR* Standardized root mean square residual

Item 11 (“Supervisor required”) and 12 (“Agency required”) in the indicators of the Requirements is fixed to 1. a = without covariance, b = with an added correlation (item 9 and 10), 3 = Bifactor model with error correlation (item 9 and 10)

**Table 4:** Standardized factor loadings from model results

EBPAS subdomains and items	Model			
	First-order (1b)	Second-order (2b)	Bifactor model (3)	
			Subdomain	General
Requirements		**0.48**		
12.Agency required	0.96	0.96	**0.87**	0.45
11.Supervisor required	0.96	0.96	**0.87**	0.37
13.State required	0.78	0.78	**0.60**	0.49
Appeal		**0.86**		
10.Make sense	0.64^a^	0.64^a^	0.31^b^	**0.55**^**b**^
9.Intuitively appealing	0.62^a^	0.63^a^	0.18^b^	**0.57**^**b**^
14.Colleagues happy	0.70	0.69	**0.57**	**0.55**
15.Enough training	0.84	0.84	0.38	**0.72**
Openness		**0.74**		
2.Will follow a manual	0.91	0.92	0.63	**0.67**
1.Like new therapy types	0.63	0.62	0.40	**0.68**
4.Research-based ok	0.82	0.82	**0.68**	0.34
8.Different from usual	0.61	0.61	0.34	0.48
Divergence		**−0.65**		
5.Research-based not useful	0.59	0.59	**0.57**	−0.36
7.Would not use manualized	0.83	0.84	0.40	**−0.57**
6.Clinical experience important	0.51	0.49	**0.54**	−0.36
3.Know better than researchers	0.38	0.39	0.35	− 0.24

*N* = 565 for all models tested. Item 11 (“Supervisor required”) and 12 (“Agency required”) in the indicators of the Requirements is fixed to 1. All models have an added correlation between item 9 and 10

For model 2b the loadings to the general factors are on the rows of the factor labels. For model 3, to highlight the items providing the best discrimination on the general factor, items loading greater than .50 on the general factor are in boldface type. Items with larger loadings on group factor than general factor are also in bold face type. ^a^residual covariance = 0.65, *p* < .001. ^b^residual covariance =0.68 *p* < .001. All factor loadings were statistically significant *p* < .001

#### First-order models

We tested the four-factor model first and found adequate fit (see Model 1a, Table 2); with the CFI and SRMR suggesting that most of the covariance was represented in this model. To further improve model fit, we fixed items 11 (“Supervisor required”) and 12 (“Agency required”) in the indicators of the Requirements factor to 1. The strongest correlation (*r* = .65) was between items #9 (“Intuitively appealing”) and #10 (“Making sense”) from the Appeal subscale, and adding this error correlation increased fit significantly (see Model 1b, Table 2). Factor inter-correlations were in the moderate range (.44). Generally loadings were high (above .5); with the exception of Divergence’s loading on item #3 (“Know better than researcher”) (.38) (see Tables 3 and 4).

#### Second-order models

A second-order model with a general factor above the four factors was tested next (see Table 2 and Fig. 1), both without (model 2a) and with the correlated items in the Appeal subscale (Model 2b). Model 2b had two more degrees of freedom than Model 1b and is therefore more parsimonious (Table 2). The CFI and SRMR suggested a good fit for Model 2b, even if the fit for this model was not as good as for Model 1b. The standardized factor loadings for the subscales were similar to the factor loadings in first order model (see Table 4). The average loading on the General Attitudes factor was also strong (.68) ranging from .48 to .86 (see Table 4 and Fig. 1).

Overall, the modification indices were difficult to interpret. For example, they suggested additional correlation between Requirements and Appeal, but also between Openness and Divergence. Adding one of these correlations increased the model fit to a level comparable to Model 1b. However, as this was not an expected correlation, we refrained from adding it to the final model.

#### Bifactor model

The bifactor model converged when the error correlation between item 9 and 10 was added and an additional item (12), from the Requirements factor was fixed to 1 (Model 3, Table 2). The item loadings on the General attitude and four subscales were significant (*p* < 0.001) and generally moderate (~.5): ranging from .24- to .72 for the General Attitudes scale; and from .18 to .87 for the subscales (see Table 4 and Fig. 2).

The explained common variance (ECV) for the General Attitudes factor was .46. The ECV for the sub-factors were: Requirement = .22; Appeal = .07; Openness = .13; and Divergence = .11. These ECVs suggest that a substantial proportion of the overall variance in the model was explained by the General Attitudes factor and the four sub-scale factors [45].

## Discussion

The present study aimed to evaluate the psychometric properties of a Swedish translation of the Evidence-Based Practice Attitude Scale (EBPAS). Overall, the Swedish version showed acceptable levels of internal consistency for the scale overall (General Attitudes) and three of the four subscales. The factor structure of the Swedish version of the EBPAS closely mimicked the structure shown for the English language original, thereby suggesting that the translation from English to Swedish did not alter how the items were interpreted and rated by the participants. The present findings provides additional support for the validity of the EBPAS in in a child and adolescent mental health contexts broadly and specifically in a Swedish context. Confirmatory factor analysis in this large and representative sample, utilizing first-order, second-order and bifactor models, provided preliminary for the proposed structure of a higher-order factor and at least three of the four domain-specific factors. The rather strong general factor supports the use of the EBPAS as a single measure of attitudes toward evidence-based practice. Most items contributed to this general factor as well as to their domain-specific factor. However, the Divergence subscale revealed a somewhat weaker correlation to the General Attitudes factor.

With respect to reliability, the Swedish version was on par with the English original, with good internal consistency for the total scale and subscales with the exception of Divergence [1, 13, 14]. One of the obvious problems with the Divergence scale is its low homogeneity and the implications for its use. Correlations between a scale with low reliability and some objective criterion (or outcome variable) will be attenuated and the statistical power will be decreased [46]*.* Another problem is that the items of the Divergence subscale are phrased in an opposite direction to the items of the other subscales and items #5 (“Researcher-based not useful”) and # 7 (“Won’t use manuals”) are negatively phrased. It has been suggested that self-rating scales should include items that are phrased in a positive and a negative way, but this may be difficult with the EPBAS. It is well-known that such differences in phrasing are associated with lower correlations between items and subscales [47]. It is important to note that the Requirement scale has consistently demonstrated strong internal consistency but consists of only three items with similar phrasing, differing by a single word [1, 14]. Similarities in item wording can impact participant responses, subsequent factor structure and may inflate a scale’s reliability. Overall the EBPAS is a reliable scale and there are obvious benefits of briefer scales when carrying out research with already over-burdened health professionals. Nevertheless, we suggest that additional items be added to all of the subscales to improve the reliability.

With respect to the factor structure, the factor loadings from the first-order model were highly similar to those found for the English language original, providing support for the validity for this Swedish version and for the four-factor model and subscale scoring more broadly [1, 13]. The Requirements subscale had the strongest loadings, followed by Openness, Appeal and Divergence.

The factor loadings in the second-order model were also similar to the English language original but loadings on the General Attitudes factor were somewhat stronger, especially for the Divergence scale [14]. Item #3 (“Know better than researchers”) had the lowest loading in this Swedish and the English language original.

One of the problems with hierarchical models is that even if they are supported they do not address whether there is unique systematic variance in the subscales [38]. Bifactor models allow a direct exploration of the extent to which subscales reflect a common target domain and the extent to which they reflect a primary subdomain. Comparing the bifactor models of the Dutch and Swedish versions of the EBPAS reveal similar loadings, but also some notable differences [32]. In line with the Dutch bifactor CFA model, the Requirements, Openness and Divergence subscales where clearly supported, but not the Appeal subscale [14]. In contrast to the Dutch study, all EBPAS items contributed to the General Attitudes factor and the unique information in the Divergence subscale was better supported. This is the first study investigating the factor structure of the EBPAS to find support for the General Attitudes factor based on a bifactor model of the scale.

Aarons has suggested that the EBPAS can be used prior to implementation efforts that aim to target specific organizational and leadership activities to enhance buy-in, subsequent uptake and sustainment of an EBP program [14]. Our findings clearly support the use of the EBPAS total score for assessing general attitudes toward EBP and the presence of multidimensionality does not handicap the ability to interpret the EBPAS as one scale. The total score includes more items than each of the four domain-specific subscales and therefore provides a larger breadth in terms of content validity and increased reliability. Previous studies find stronger evidence of convergent validity for the EBPAS total score than for the subscales but somewhat weaker evidence in relation to the total score’s predictive validity [15–18]. It is important to point out that in any study, a *drawback of a using a total score is that you lose information about the relation between the individual facets measured by the scale and a criterion variable* [46]*.* In addition, if only some of the facets predict an expected outcome, the total scale will result in weaker predictions than the subscale themselves. If the different facets are related to an implementation outcome in an opposite direction, it can also result in a misleading null-finding. Our results suggest that the Requirement, Openness, but possibly not the Appeal and Divergence subscales can be scored and used as indicators of specific facets of attitudes towards EBP when a more precise and specific analysis is needed. However, implementation project planners and researcher should be aware that a strong relationship between the subscales and any implementation outcome may be due to the common variance (i.e., general attitude) measured by the subscale and not its unique contribution to the implementation outcome. More importantly, until unique contribution is better supported by adding items to increase the reliability of the EBPAS subscales, we cannot recommend that the subscales be used as predictors of implementation outcomes independently of the total scale.

Additional studies are needed that examine the factor structure of the Swedish version of the EBPAS in different healthcare contexts (e.g., with adult mental health professionals or healthcare professionals more broadly). Likewise further studies are needed that examine the relationship between the EBPAS (subscales and total scale) and different outcome variables, including the EBPAS’ ability to predict the adoption, fidelity to and sustainment of EBP in a Swedish context.

Nevertheless, the present results should be taken into consideration in any future revisions of the EBPAS. The original (English language) EBPAS has previously been modified, retaining the 15 original items and subscales and adding 35 items covering eight new domains of attitudes towards EBP [47]. This 50-item version has been shortened to 36 items; keeping the 12 domain-specific subscales but with one item removed from each of the four-item subscales, including Items #9 (Appeal),#8 (Openness), and #3 (Divergence) [48]. The subscales in these revised versions have received preliminary support but use of the total score as a single scale requires further validation [47–49]. Based on the results from the present and previous research with the 15-item EBPAS, we suggest a somewhat different approach to further revision, namely the development and evaluation of the briefer version: replacing the problematic Item #3 from the Divergence scale, rewording items in the Requirement subscales to make them less similar, replacing items from the Appeal subscale that are too similar, and adding a few items to the four original domain-specific subscales to increase their reliability. In this way it may be possible to retain a relatively brief scale that is valid and reliable, and practical for use in routine care settings where clinicians often complain of being overburdened with forms to complete.

An issue of relevance to the further development of measures of EBP broadly, and the EBPAS specifically, concerns the content validity of the individual items. Conceptions of EBP have developed over time in the literature and so it is likely that what practitioners understand as the “behavioral” components of EBP, as well as attitudes, is likely to change over time and vary between different healthcare contexts. In their effort to develop a theory- and data-driven -focused approach to item development for a measure of EBP that could be used in an implementation context, Burgess et al. suggested that inclusion of items that measure the importance of clinical experience over EBPs, clinician openness to change and problems with EBPs would increase the pragmatic utility of future measures [15].

Findings from the present study should be viewed within the context of certain methodological strengths and limitations. The sample represents front-line practitioners from a geographically diverse area covering more than a third of Sweden’s child and adolescent mental health services and representing more than half of the Swedish regions (counties). The sample’s characteristics were similar to available national data describing the child mental health service workforce and sufficiently robust in size for the purposes of estimating internal reliability and carrying out confirmatory factor analyses [50]. We were unable to obtain data from non-respondents to the survey and that would have allowed us to examine potential bias. Also, the likelihood that non-responders should have influenced the correlation between the items, investigated here, is less likely; than it should have influenced the mean levels.

The data originated from 11 different services, but the difference between them were rather small and could not have had any decisive influence on the estimations. Finally, there was insufficient sample size to permit creation of meaningful subgroups of participants to allow testing for invariance between groups. Invariance tests should be conducted in future validation studies of the Swedish EPBAS.

## Conclusions

The present study provides support for the reliability and construct validity of the Swedish version of the EBPAS. The internal consistency coefficients for the subscales and scale overall, and the observed factor structures of the Swedish version were comparable to those reported for the English language original and other language translations. The EBPAS total score can be used as a measure of global attitudes toward EBP in implementation project planning and research, but the subscales should only be used in conjunction with the total score. Further revision of the EBPAS is warranted in order to improve the reliability and validity of the subscales.

## Supplementary information

**Additional file 1.** Results of Prior Confirmatory Factor Analyses. Aaron’s previous factor analytic results and van Sonsbeek bifactor model result (sample size, model fit and factor loadings)

## Data Availability

The dataset supporting the conclusions of this article is available in the Halland Hospital Halmstad repository. The dataset used and/or analyzed during current study are available from the corresponding author on reasonable request.

## Abbreviations

EBP: Evidence-based practice
EBPAS: Evidence-based practice attitude scale
CAMHS: Child and adolescent mental health services
CFA: Confirmatory factor analysis
WLSMV: Weighted least squares -robust mean and variance adjusted
RMSEA: Root mean square error of approximation
CFI: Comparative fit index
TLI: Tucker-lewis index
SRMR: Standardized root mean square residual
WRMR: Weighted root mean square residual
