# Supplementary material for: Confirmatory factor analysis of the Evidence-Based Practice Attitude Scale (EBPAS) in a large and representative Swedish sample: is the use of the total scale and subscale scores justified?
Source: BMC Med Res Methodol. 2020 Oct 14;20:254. doi: 10.1186/s12874-020-01126-4 (PMC7557010; doi:10.1186/s12874-020-01126-4)
Supplement: Supplementary file 1 — Additional file 1. Results of Prior Confirmatory Factor Analyses. Aaron’s previous factor analytic results and van Sonsbeek bifactor model result (sample size, model fit and factor loadings) [file 12874_2020_1126_MOESM1_ESM.docx]

| **Additional Table 1** Results of Prior Confirmatory Factor Analyses | | | | | |
| --- | --- | --- | --- | --- | --- |
| Sample | Aarons  (2004) | Aarons et al  (2007) | Aarons, Glisson et al  (2010) | Van Sonsbeek  (2015) | |
| Respondents | 163 | 221 | 1089 | 270 | |
| Model | First-order  (1a) | First-order  added covariance  (1b) | Second-order added covariance  (2b) | Bifactor model  (3a) | |
| Fit statistics |  |  |  |  | |
| Chi square | 144.92 (84), | 183.51(83) | 409.72 (85) | 107,37(75) | |
| CFI/TLI | .93/.92 | .92/90 | .94/.93 | .97/.96 | |
| RMSEA | .067 | .07 | .059 | .004 | |
| Factor loadings |  |  |  | Subdomain | General |
| Requirements |  |  | **0.44** |  |  |
| 12.Agency required | 0.99 | 0.99 | 0.99 | 0.89 | 0.44 |
| 11.Supervisor required | 0.88 | 0.90 | 0.88 | 0.74 | 0.40 |
| 13.State required | 0.78 | 0.81 | 0.77 | 0.64 | 0.40 |
| Appeal |  |  | **0.89** |  |  |
| 10.Make sense | 0.89 | 0.46 ^a^ | 0.63^b^ | 0.75 | 0.45 |
| 9.Intuitively appealing | 0.83 | 0.39 ^a^ | 0.49^b^ | 0.59 | 0.47 |
| 14.Colleagues happy | 0.56 | 0.57 | 0.75 | *0.20* | 0.54 |
| 15.Enough training | 0.55 | 0.57 | 0.80 | *0.21* | 0.54 |
| Openness |  |  | **0.61** |  |  |
| 2.Will follow a treatment m | 0.61 | 0.61 | 0.78 | 0.43 | 0.56 |
| 1.Like new therapy types | 0.62 | 0.37 | 0.70 | 0.56 | 0.33 |
| 4.Research-based ok | 0.81 | 0.56 | 0.68 | 0.44 | 0.55 |
| 8.Different from usual | 0.66 | 0.51 | 0.613 | 0.36 | 0.60 |
| Divergence |  |  | **-0.22** |  |  |
| 5.Research-based not useful | 0.65 | 0.38 | 0.68 | 0.64 | -0.18 |
| 7.Would not use manualized | 0.76 | 0.43 | 0.57 | 0.56 | -0.27 |
| 6.Clinical experience imp. | 0.42 | 0.37 | 0.55 | 0.60 | *0.02* |
| 3.Know better than researchers | 0.34 | 0.40 | 0.49 | 0.50 | *-0.08* |
| a =without added error correlation b= with added e rror correlation (item 9 and 10), 3= Bifactor model without added error correlation. CFI= Confirmatory fit index, TLI=Tucker-Lewis index, RMSEA=root mean square approximation, SRMR= standardized root mean square residual..Subdomain= item loading on sub-factor, General=item loading on general factor. All factor loadings are significant except #6 and #3 on general factor and #14 and #13 on the Appeal factor in the bifactor model . Non -significant factor loadings are in italics. For model 2b the loadings to the general factors are on the rows of the factor labels. ^a^residual covariance=freel yestimated  ^b^residual covariance= 0.60, p<.001. | | | | | |
